# Identification of PLOD Family Genes as Novel Prognostic Biomarkers for Hepatocellular Carcinoma

**DOI:** 10.3389/fonc.2020.01695

**Published:** 2020-09-04

**Authors:** Bo Yang, Yonghui Zhao, Lan Wang, Yuanyuan Zhao, Lai Wei, Dong Chen, Zhishui Chen

**Affiliations:** ^1^Institute of Organ Transplantation, Tongji Hospital, Tongji Medical College, Huazhong University of Science and Technology, Wuhan, China; ^2^Key Laboratory of Organ Transplantation, Ministry of Education, Ministry of Public Health, Chinese Academy of Medical Sciences, Wuhan, China; ^3^Department of Neurosurgery, Cangzhou Central Hospital, Cangzhou, China; ^4^Reproductive Medicine Center, Tongji Hospital, Tongji Medical College, Huazhong University of Science and Technology, Wuhan, China

**Keywords:** Hepatocellular carcinoma, PLOD, prognostic biomarker, extracellular matrix, tumor-infiltrating immune cells

## Abstract

**Background:**

Hepatocellular carcinoma (HCC) is one of the most common malignancies with rising incidence and persistently high mortality. Previous researches have demonstrated that some *PLOD* family members are associated with tumor progression and metastasis in most human cancers. However, the prognostic and biological roles of *PLODs* in HCC remain largely unknown.

**Methods:**

ONCOMINE, HPA, UALCAN, GEPIA, cBioPortal, GeneMANIA, NetworkAnalyst, Metascape, DAVID 6.8, and TIMER were used to determine the prognostic values and biological function of *PLOD* family members in HCC.

**Results:**

The mRNA and protein expression patterns of *PLOD* family members were noticeably upregulated in HCC compared to normal tissue. The high expression levels of *PLOD1* and *PLOD2* genes were significantly correlated with higher tumor grades in HCC patients. In addition, the high expression levels of *PLOD1–3* were remarkably associated with poor overall survival in HCC patients, while high *PLOD1* and *PLOD3* expression were markedly associated with worse disease-free survival. In the co-expression gene analysis, 20 genes were primarily associated with the differentially expressed *PLOD* family members in HCC cases. Through functional enrichment analysis, the biological functions of *PLODs* in HCC were mainly involved in collagen fibril organization, lysine degradation, collagen biosynthesis, and modifying enzymes. Furthermore, the expression levels of *PLOD1–3* were positively correlated with the activities of tumor-infiltrating immune cells, including macrophages, neutrophils, CD4+ T cells, and dendritic cells. Besides, the expression levels of *PLOD2* and *PLOD3* were positively correlated with the infiltrating levels of B cells.

**Conclusion:**

The findings of this study could provide novel insights into the identification of prognostic biomarkers for HCC patients.

## Introduction

Liver cancer is the seventh most common form of cancer worldwide and the second leading cause of cancer-related mortality (1). Hepatocellular carcinoma (HCC), accounting for over 75%, is the predominant histologic type of liver cancer (2). The molecular mechanism of HCC formation and progression remains largely unclear, thus hindering the effective treatment of the disease (3). Recently, growing evidence has suggested that the elevated deposition of collagen and its cross-linking can worsen tumor progression by promoting cancer cell proliferation, migration, and invasion (4, 5). Collagen deposition and cross-linking are dependent on the hydroxylation of lysine residues, which is mainly catalyzed by procollagen-lysine, 2-oxoglutarate 5-dioxygenase (PLOD). Three lysyl hydroxylase isoforms (LH1-3) have been characterized thus far, namely: PLOD1, PLOD2, and PLOD3 (6). The expression levels of PLOD1–3 (PLOD1, PLOD2, and PLOD3) are controlled by multiple cytokines, transcription factors, and miRNA species (7). Abnormal expression of *PLOD1–3* genes can promote tumor progression and metastasis, and it is reasonable to speculate that PLODs are the potential targets for HCC treatment (8). However, little is known about the expression patterns and functional roles of *PLOD* in HCC prognosis. This study aimed to evaluate the biological functions and prognostic roles of *PLOD1–3* genes in human HCC tissues.

## Materials and Methods

### ONCOMINE Data Analysis

ONCOMINE^1^ provides a wide range of translational bioinformatic services for the analysis of genome-wide expression (9). The expression data of *PLOD* family genes (i.e., *PLOD1*, *PLOD2*, and *PLOD3*) in various types of cancers were identified from ONCOMINE database using the analysis of “Gene summary view” and “Dataset view”; *P* values were obtained using the Student’s *t* test. The parameters were set as follows: *P* value < 0.05; FC, >2; and gene ranking, top 10%. Analysis type: cancer vs. normal analysis; data type: mRNA. Cancers, genes, datasets, sample sizes, fold change, *t* test, and *P* value were obtained from studies that showed statistical differences.

### Human Protein Atlas (HPA) Data Analysis

Human Protein Atlas (HPA)^2^ is a user-friendly website that comprises immunohistochemistry-based expression profiles for the top 20 most common forms of cancer (*n* = 12 for each cancer type) (10). The protein expression levels of *PLOD* family members in normal and HCC tissues were assessed using the database. According to the fraction of stained cells, staining quantity was also divided into four levels: none, <25%, 25–75%, and >75%. Protein expression levels were based on staining intensity and staining quantity. The classification criteria for protein expression levels were as follows: negative, not detected; weak and <25%, not detected; weak combined with either 25–75% or 75%, low; moderate and <25%, low; moderate combined with either 25–75% or 75%, medium; strong and <25%, medium; and strong combined with either 25–75% or 75%, high.

### UALCAN Data Analysis

UALCAN^3^ is a comprehensive web tool that provides analysis according to both MET500 cohort and the Cancer Genome Atlas (TCGA) datasets (11). In this work, we analyzed the relative expression of *PLOD1–3* across normal and cancer tissues, as well as among various tumor sub-groups based on cancer stage. Student’s *t* test was employed to compare the statistical difference between two groups. *P* value < 0.05 was deemed as statistically significant.

### GEPIA Data Analysis

GEPIA^4^ is an interactive web that covers 8587 normal and 9736 cancer tissues from both TCGA and Genotype-Tissue Expression (GTEx) projects (12). In this study, we examined the expression of *PLOD* family members in 529 patients from TCGA database with LIHC, between low- and high-expression groups based on gene expression by using the logrank and Mantel–Cox tests. A total of 584 patients were used to evaluate the overall survival (OS) and disease-free survival (DFS) of patients at risk. Hazard ratio (HR), 95% confident interval (CI), and *P* value were calculated accordingly.

### cBioPortal Data Analysis

The cBioPortal for Cancer Genomics^5^ is a comprehensive web resource that can visualize and analyze multidimensional cancer genomic data (13). Copy number variation (CNV), mutations, and the summary of the gene types in HCC were evaluated according to the online instructions of cBioPortal. In addition, the relationship between gene mutation and HCC prognosis was analyzed using the cBioPortal tool based on TCGA database. The *P* value set as 0.05 was considered significantly different.

### GeneMANIA Data Analysis

GeneMANIA^6^ is a prediction server for analyzing genetic and protein interactions, co-expression, pathways, co-localization, and domain-protein similarity of target genes (14). In the present study, we analyzed the relationship between *PLOD* family members and their interactive genes by using this database.

### NetworkAnalyst Data Analysis

NetworkAnalyst^7^ is an analytics platform that integrates tissue- or cell-type-specific protein–protein interaction (PPI) network, gene co-expression network, and gene regulatory network (15). In this study, we constructed the gene–gene network of *PLOD* family members and co-expression genes by using the NetworkAnalyst tool.

### Metascape Analysis

Metascape^8^ is an intuitive software for gene annotation and gene set enrichment analysis (16). In this study, we assessed the functions of *PLOD* family members and their co-expression genes. The *P* value was set as 0.01, and the enrichment factor of >1.5 and minimum count of 3 were considered as significant.

### DAVID Analysis

DAVID^9^ contains a comprehensive set of functional annotation tools for better clarifying the biological functions of target genes (17). In this work, Gene Ontology (GO) and Kyoto Encyclopedia of Genes and Genomes (KEGG) pathway enrichment analyses of *PLOD* family members and their closely related neighbor genes were conducted using DAVID tool. The cutoff value for significant GO terms and KEGG pathways was a false discover rate (FDR) of <0.05.

### TIMER Database Analysis

TIMER^10^ is an intuitive software that can be used to systematically evaluate the infiltration of various immune cells and their clinical impacts (18). In this study, we assessed the expression levels of *PLOD* family members in HCC and their correlations with tumor purity and infiltrating immune cells such as B cells, macrophages, neutrophils, CD4+ T cells, CD8+ T cells, and dendritic cells.

## Results

### Aberrant Expression of PLOD Family Genes in HCC Patients

**FIGURE 1:**
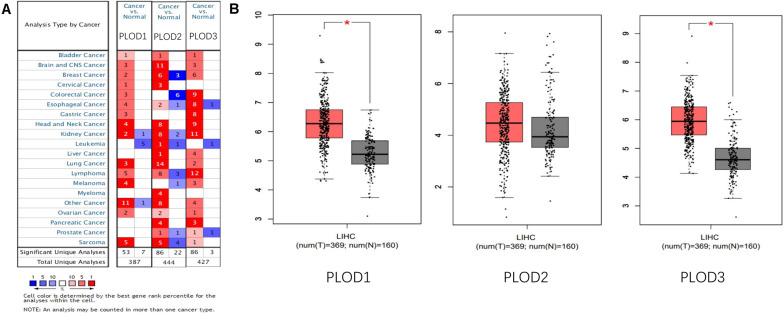
The expression of PLOD family members in different tissues. **(A)** The transcription levels of PLOD family members in different types of cancers Red, upregulation; blue, downregulation. The number in each cell indicates the datasets that met the set threshold in each cancer type. Cell color was defined as the gene rank percentile for analyses within the cell. **(B)** Boxplot results of the expression levels of PLODs family members in HCC analyzed using GEPIA. Red box, tumor samples; gray box, normal samples. T, tumor; N, and normal. * means *P* < 0.05.

Information on *PLOD* family genes (i.e., *PLOD1*, *PLOD2*, and *PLOD3*) was obtained from the ONCOMINE database. Next, the expression levels of the three *PLOD* family genes were compared between 20 different types of human cancers and adjacent normal tissues. According to the ONCOMINE data, the hepatic expression levels of *PLOD2* and *PLOD3* were remarkably elevated in liver cancer tissue compared to normal liver tissue (Figure 1A). In addition, the expression levels of PLOD family genes in HCC and control tissues were compared by using GEPIA. The results showed that the transcriptional levels of *PLOD1 and PLOD3* (*P* < 0.05) were significantly elevated in HCC tissue compared with normal tissue (Figure 1B).

**FIGURE 2:**
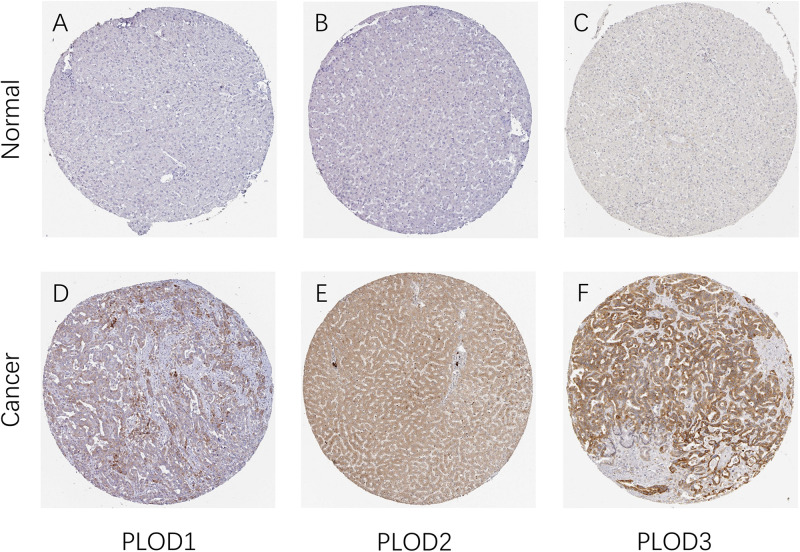
The protein expression of PLOD family members in patients with HCC. **(A,D)** Representative immunohistochemistry images of PLOD1 in HCC and non-cancerous liver tissues derived from the HPA database. **(B,E)** Representative immunohistochemistry images of PLOD2 in HCC and non-cancerous liver tissues derived from the HPA database. **(C,F)** Representative immunohistochemistry images of PLOD3 in HCC and non-cancerous liver tissues derived from the HPA database. HPA, Human Protein Atlas.

After determining the transcriptional levels of the three *PLOD* members, the protein levels of these *PLOD* members in HCC patients were analyzed using the HPA database. Noteworthy, the higher expression levels of *PLOD1–3* proteins were observed in HCC tissues than normal. As shown in Figure 2, the protein expression of PLOD1 was upregulated in HCC with medium staining. In comparison, the protein expression of *PLOD2* and *PLOD3* was obviously upregulated in HCC with strong staining. Taken together, these findings indicate that the three *PLOD* family genes are overexpressed in HCC patients at both mRNA and protein levels.

### The Prognostic Values of PLOD Family Members in HCC Patients

**FIGURE 3:**
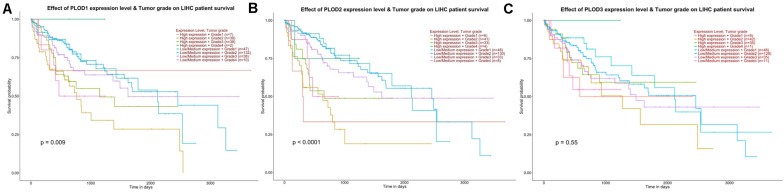
The effect of PLOD family expression level for the tumor grade on HCC patient survival. **(A)** The overexpression of PLOD1 has the poorest prognosis in higher tumor grade of HCC patients. **(B)** The overexpression of PLOD2 has the poorest prognosis in higher tumor grade of HCC patients. **(C)** The differential expression levels of PLOD3 were not significant correlation with the tumor grade of HCC patients.

To identify the *PLOD* members related to carcinogenesis, progression, and prognosis of HCC, the differential expression levels of *PLOD* members were correlated with the tumor grade of HCC patients using ULCAN. The remarkable association between *PLOD1* (*P* = 0.009) and *PLOD2* (*P* < 0.0001) expressions and tumor grade was observed (Figure 3). As HCC progressed, the expression levels of *PLOD1* and *PLOD2* were also increased. These findings indicate that these *PLOD* members play crucial roles in the carcinogenesis and progression of HCC.

**FIGURE 4:**
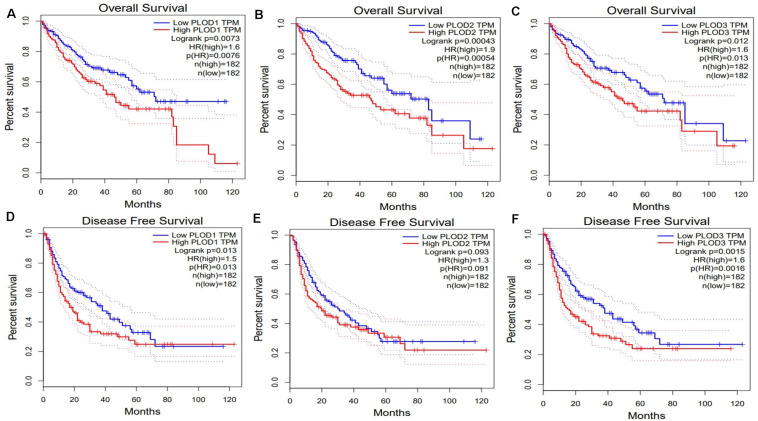
The survival analysis of PLOD family members in HCC. **(A–C)** Overexpression levels of PLOD1, PLOD2, and PLOD3 were associated with shorter OS in HCC. **(D–F)** Overexpression levels of PLOD1and PLOD3 were associated with shorter DFS in HCC, except PLOD2.

Next, we evaluated the association between differentially expressed *PLOD* members and HCC prognosis using the GEPIA database. The OS curves of the three *PLOD* members are demonstrated in Figures 4A–C, respectively. Notably, high transcriptional levels of *PLOD1* (*P* = 0.0073), *PLOD2* (*P* = 0.00043), and *PLOD3* (*P* = 0.012) were markedly associated with shorter OS in HCC patients. The prognostic roles of differentially expressed *PLOD* members in the DFS of HCC patients were also explored. It was found that high transcriptional levels of *PLOD1* (*P* = 0.013) and *PLOD3* (*P* = 0.0015) were remarkably associated with shorter DFS in HCC patients (Figures 4D,E).

### Alteration in the Frequency of PLOD Family Genes in HCC Patients

**FIGURE 5:**
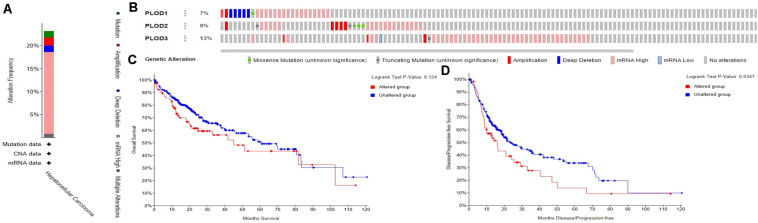
Alteration frequency of PLOD family members and their survival analysis in LICH (cBioPortal). **(A)** Summary of alterations in PLOD family members in LICH. **(B)** OncoPrint visual summary of PLODs alterations. **(C)** Kaplan–Meier plots comparing OS in cases with/without PLOD family member gene alterations in LICH. **(D)** Kaplan–Meier plots comparing disease-free survival (DFS) in cases with/without PLOD family member alterations in LICH.

The frequency of genetic alterations in the three *PLOD* genes among HCC patients were identified using the cBioPortal database. As shown in Figure 5A, 84 (23%) HCC patients exhibited significant alterations in the three *PLOD* genes, including amplification, deep deletion, truncating mutation, missense mutation, and transcriptional upregulation. Specifically, the percentage changes in the genetic alterations of *PLOD1*, *PLOD2*, and *PLOD3* among HCC patients were 7, 8, and 13%, respectively, (Figure 5B). Furthermore, we then examined the association between the changes in *PLOD* gene expression and HCC prognosis through the cBioPortal database. Kaplan–Meier curves were used to determine the OS and DFS of HCC patients with altered or unaltered mRNA expression levels of *PLOD1*–*3*. The results demonstrated that the alteration of *PLOD* genes in HCC patients was not remarkably associated with OS (*P* = 0.124; Figure 5C). However, HCC patients with altered *PLOD* gene expression exhibited a markedly worse DFS (*P* = 0.0347) compared to those with unaltered *PLOD* gene expression (Figure 5D).

### Co-expression and Functional Enrichment Analysis of PLOD Family Genes in HCC Patients

**FIGURE 6:**
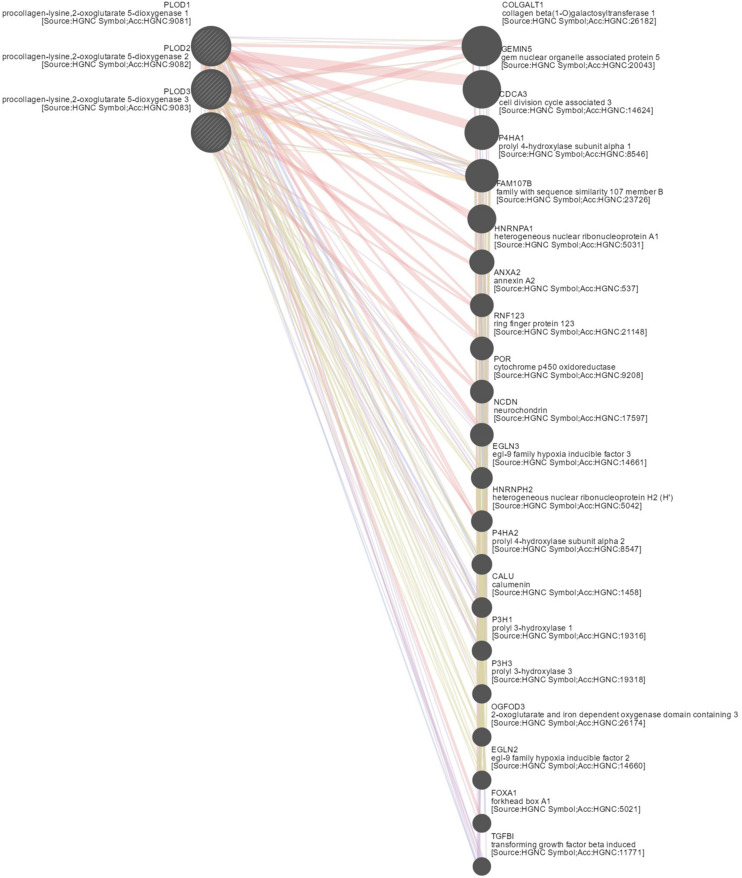
Gene–gene network of PLOD family members in LICH (GeneMANIA). GeneMANIA was used to construct a network of PLODs and their functionally related genes. The database identified 20 genes that were closely associated with PLODs.

To explore the underlying mechanism of *PLOD* family members in HCC, we constructed a network of *PLOD* family members and their functionally related genes using GeneMANIA and NetworkAnalyst. The results showed that 20 genes, such as *TGFBI*, *FOXA1*, *EGLN2*, *OGFOD3*, *P3H3*, *P3H1*, *CALU*, *P4HA2*, *HNRNPH2*, *EGLN3*, *NCDN*, *POR*, *RNF123*, *ANXA2*, *HNRNPA1*, *FAM107B*, *P4HA1*, *CDCA3*, *GEMIN5*, and *COLGALT1*, were mainly related to the regulatory functions of differentially expressed *PLOD* family genes in HCC patients (Figure 6).

**FIGURE 7:**
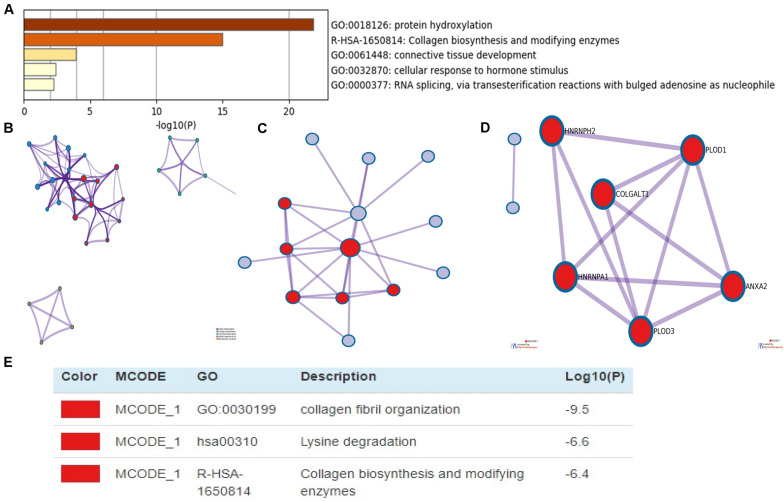
The enrichment analysis of PLODs and the 20 most co-expressed genes in patients with LIGH (Metascape). **(A)** Bar graph of top 5 enriched terms for PLODs and the 20 most co-expressed genes. **(B)** Network of enriched terms, colored by cluster ID. **(C–E)** PPI network and MCODE components identified.

DAVID and Metascape were used to assess the biological function of differentially expressed *PLOD* family and the above co-expressed genes. The data revealed the top 5 most enriched terms, including protein hydroxylation, collagen biosynthesis and modifying enzymes, connective tissue development, cellular response to hormone stimulus, and RNA splicing, via transesterification reactions with bulged adenosine as nucleophile (Figure 7A). Additionally, we constructed the network of enriched terms colored by ID (Figure 7B). To further explore the association between differentially expressed *PLOD* family genes and HCC, a PPI network was constructed and mCODE analysis was carried out. Moreover, the most significant mCODE components were extracted from the protein–protein interactive network, and the results showed that biological processes were primarily related to collagen fibril organization, lysine degradation and collagen biosynthesis, and modifying enzymes (Figures 7C–E).

### Immune Cell Infiltration of PLOD Family Genes in HCC Patients

**FIGURE 8:**
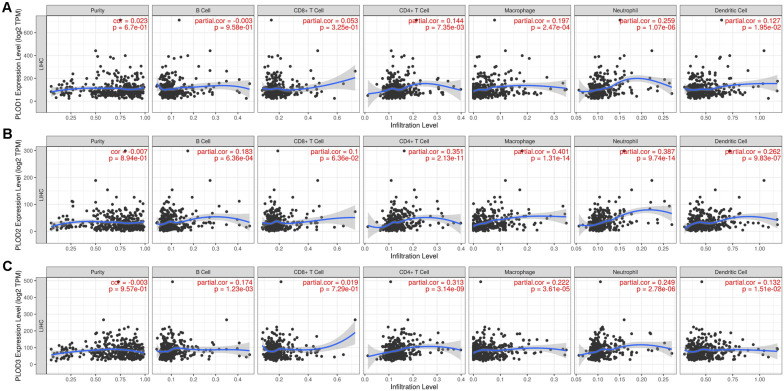
Correlations of PLODs expression with immune infiltration level in LIGH. **(A)** The correlation between each type of TIICs (B-cells, CD4+ T-cells, CD8+ T-cells, neutrophils, macrophages, and dendritic cells) and PLOD1. **(B)** The correlation between each type of TIICs (B-cells, CD4+ T-cells, CD8+ T-cells, neutrophils, macrophages, and dendritic cells) and PLOD2. **(C)** The correlation between each type of TIICs (B-cells, CD4+ T-cells, CD8+ T-cells, neutrophils, macrophages, and dendritic cells) and PLOD3.

Considering the fact that inflammatory response and infiltrating immune cells can affect the prognosis of HCC, we also evaluated the association between differentially expressed *PLOD* family genes and immune cell infiltration using the TIMER database. As shown in Figure 8A, a positive correlation was observed between *PLOD1* expression and the infiltrating CD4+ T cells (Cor = 0.144, *P* = 7.35e-3), macrophages (Cor = 0.197, *P* = 2.47e-4), neutrophils (Cor = 0.259, *P* = 1.07e-6), and dendritic cells (Cor = 0.127, *P* = 1.95e-2). *PLOD2* expression was negatively correlated to the infiltrating B cells (Cor = -0.183, *P* = 6.36e-4), while positively correlated to the infiltrating CD4+ T cells (Cor = 0.351, *P* = 2.13e-11), macrophages (Cor = 0.401, *P* = 1.31e-14), neutrophils (Cor = 0.387, *P* = 9.74e-14), and dendritic cells (Cor = 0.262, *P* = 9.83e-7; Figure 8B). Similarly, *PLOD3* expression was negatively correlated to the infiltrating B cells (Cor = -0.174, *P* = 1.23e-3), while positively correlated to the infiltrating CD4+ T cells (Cor = 0.313, *P* = 3.14e-9), macrophages (Cor = 0.222, *P* = 3.61e-5), neutrophils (Cor = 0.249, *P* = 2.87e-6), and dendritic cells (Cor = 0.132, *P* = 1.51e-2; Figure 8C).

## Discussion

In this work, our results indicated that *PLOD1–3* were highly expressed in HCC. We further proved that the high expression levels of *PLOD1* and *PLOD2* were markedly correlated with higher tumor grade. In addition, *PLOD1–3* overexpressions were associated with shorter OS, while *PLOD1* and *PLOD3* overexpressions were associated with shorter DFS in HCC patients. To date, increasing evidence has suggested the significant roles of *PLOD2* and *PLOD3* in HCC. Previous research has found that *PLOD2* expression is remarkably correlated to tumor size and DFS in HCC patients (19). Other studies have demonstrated that *PLOD3* is overexpressed in HCC and may be a potential diagnostic biomarker for early-stage HCC (19, 20). Furthermore, a recent study has indicated that *PLOD3* knockdown can suppress tumor growth in the liver of spontaneous HCC mice. Our findings of *PLOD* family genes in HCC were consistent with those reported previously (20). Thus, it is speculated that individual *PLOD* gene or *PLOD* family genes may serve as potential prognostic biomarkers for HCC patients. However, the functional roles of *PLOD* family members in HCC tumorigenesis, metastasis, cell proliferation, and apoptosis have not been well-characterized. There are several limitations in our study. First, we used data from multiple different databases, and it is hard to guarantee the unity among different databases. Second, we did not verify the data from these databases. This will be done in our future study.

Previous studies have revealed that the mechanism of *PLOD* family members in other diseases is mainly attributed to its regulation of numerous signaling pathways (21). In this research, we explored the core genes that are potentially associated with *PLOD* function, and some of them have been identified as an important regulator of *PLODs*. Gjaltema et al. (22) have reported that *PLOD2* expression can be upregulated by interacting with the *TGF-*β*1* signaling-related transcription factors *SMAD3* and *SP1* and is associated with the increased acetylation of H3 and H4 histones on its gene promoter region. In addition, *FOXA1* transcription factor has been identified as a potential regulator of *PLOD2* expression during the progression of non-small cell lung cancer (23). In the current research, we further performed functional enrichment analysis to understand the biological functions of *PLOD* in HCC, and the results showed that these genes were mainly involved in collagen fibril organization, lysine degradation, collagen biosynthesis, and modifying enzymes. Collagen is recognized as the most important component of extracellular matrix, which provides multiple biochemical and biophysical cues to tumor cells (24). Moreover, abnormal lysine degradation is involved in the progression of collagen-related diseases, including cancer, and fibrosis (25, 26). Hence, the exact mechanism of *PLODs* in HCC patients still warrants further investigation.

Numerous recent studies have shown that a systematic evaluation of tumor-infiltrating immune cells (TICs) is of great importance to predict clinical outcome and develop immunotherapy (27). Nataliya et al. assessed the relative proportions of immune cells in healthy human livers and HCC or HCC adjacent tissues by applying CIBERSORT and found that resting mast cells, total and naïve B cells, and CD4+ memory resting and CD8+ T cells were increased in HCC, while activated mast cells, monocytes, and plasma cells were decreased when compared to healthy livers. Different molecular HCC subclasses with different prognosis have different immune microenvironment, and M1-type macrophages are the most representative (28). However, there is little information available about the prognostic roles of TICs in HCC patients and their potential effectiveness for immunotherapy. At present, few studies have suggested the prognostic importance of TICs and other immune molecules, including tumor-associated macrophages, natural killer cells, dendritic cells, TIM-3, PD-L1, and PD-L2 in HCC patients (29–32). So, we analyzed the infiltration rates of various immune cells (B cell, CD8+ T cell, CD4+ T cell, macrophage, neutrophil, and dendritic cell) with different expression levels of *PLOD* genes. Interestingly, we observed that the expression levels of *PLOD* genes were noticeably correlated with immune cell infiltration, which may help us gain deeper insights into the immune landscape of HCC. In addition, the overall findings of *PLOD* family members provide evidence for clinicians to predict the survival rates of HCC patients. Nevertheless, further investigations are needed to verify the robustness of these biomarkers.

In summary, our findings demonstrated a significant correlation among *PLOD* gene expression, tumor prognosis, and cancer immune microenvironment, indicating that *PLOD* family members may mediate tumor progression and exert immunotherapeutic effects on HCC. Thus, a better understanding of how this gene is regulated during tumor progression may pinpoint the potential prognostic and therapeutic roles of *PLODs* in HCC.

## Data Availability Statement

The datasets presented in this study can be found in online repositories. The names of the repository/repositories and accession number(s) can be found in the article/supplementary material.

## Author Contributions

BY and YoZ contributed to the study concept and design of this study. LWa, YuZ, and LWe contributed to the acquisition, analysis, interpretation of data, and the drafting of the manuscript. DC and ZC contributed to the review and the revision of the manuscript. All authors gave final approval to this manuscript for publication.

## Conflict of Interest

The authors declare that the research was conducted in the absence of any commercial or financial relationships that could be construed as a potential conflict of interest.

## Footnotes

1 http://oncomine.org

2 http://proteinatlas.org

3 http://ualcan.path.uab.edu/analysis.html

4 http://gepia.cancer-pku.cn/

5 https://cbioportal.org

6 http://www.genemania.org

7 https://www.networkanalyst.ca/

8 http://metascape.org

9 https://david.ncifcrf.gov/home.jsp

10 https://cistrome.shinyapps.io/timer/
